# Light distribution at the fruit tree-crop interface and consequences for yield in sloping upland agroforestry

**DOI:** 10.1016/j.heliyon.2024.e38655

**Published:** 2024-09-29

**Authors:** Huu Thuong Pham, Nguyen La, Ingrid Öborn, Göran Bergkvist, Rachmat Mulia, Sigrun Dahlin

**Affiliations:** a Department of Crop Production Ecology, Swedish University of Agricultural Sciences (SLU), Sweden; b World Agroforestry (CIFOR-ICRAF Vietnam) Viet Nam

**Keywords:** Agroforestry, Incident light, Light distribution, Light interception, Sloping land

## Abstract

Agroforestry can improve soil conservation and overall farm productivity compared with sole-crop systems, but its benefits are limited by competitive interactions between tree and crop components. Studies on light competition have been performed on relatively flat land, but slope can influence light distribution. Little is known about optimizing light utilization and enhancing system productivity and/or income from agroforestry on sloping land.

This study examined how slope influences light distribution and performance of maize and coffee crops in fruit tree-crop agroforestry. Starting hypotheses were that 1) crops upslope of tree rows receive and intercept greater amounts of light than those downslope; and 2) position of the crop is more important for light interception and yield when fruit trees have a large, dense canopy.

Five-year-old fruit-crop agroforestry experiments on west-southwest facing slopes were revisited. Each agroforestry treatment was divided into nine zones relative to the tree rows (zone 5), with zones 1–4 upslope and 6–9 downslope of the fruit tree row. Light distribution was assessed using Hemiview and SunScan and compared with that in sole-maize and sole-coffee systems. Crop growth and yield were also recorded.

Incident light to the crop was higher in the sole-crop system than in agroforestry. In agroforestry, incident light to the crops was lower downslope of trees than upslope but increased with increasing distance from the tree rows. On average, 0.40–0.50 fraction of total light reached the soil surface. Downslope had a stronger negative effect on light distribution and crop yield than upslope. The available light at the soil surface provides scope for additional components. Further studies on the light demands of different crops during the season could improve system design.

## Highlights

• Agroforestry intercepted a higher proportion of light than sole crops.
• Light distribution was mainly affected at the downslope side of the tree row.
• Maize performance correlated with the intercepted light, but coffee did not.
• Light reached the soil surface, so further component(s) could be added.

**Table:** Abbreviations

AF	Agroforestry
Longan-AF	Longan-maize agroforestry sub-treatment
Mango-AF	Mango-maize agroforestry sub-treatment
Fruit-maize-AF	Fruit tree-maize-grass agroforestry
Fruit-coffee-AF	Fruit tree-coffee-grass agroforestry
LAI	Leaf area index
SM	Sole maize
SC	Sole coffee
SS1	SunScan probe
BF5	Sunshine sensor
PDA	Handheld computer
D10	Tree trunk diameter at 10 cm height above the ground
D15	Coffee stem diameter at 15 cm height above the ground
LA	Plant leaf area

## 1 Introduction

Sloping uplands play a vital role in sustainable development of the global economy to meet human food demand and reduce poverty [1,2], with rapid population increase creating pressure to expand agriculture onto sloping land. Sloping uplands are especially important in tropical regions, where they account for approximately 50 % of total land area [3]. Shifting cultivation has been the traditional use of sloping land for annual crop production, but population growth, land use laws, and lack of suitable land are leading to shorter fallow periods or continuous cultivation. Farmers on sloping land are facing serious problems, e.g., soil erosion, nutrient depletion, water shortage, some of which are accentuated by climate change [1] and limited road access [4]. These problems are affecting agricultural productivity, farmers’ livelihoods, and sustainable development of communities on sloping land.

Agroforestry (trees on farms and in agricultural landscapes) can increase and diversify farm production and income, increase productivity, and preserve the environment [[5], [6], [7]]. The benefits of agroforestry derive from the combined interaction of many factors over the long-term [8]. They include higher biodiversity [9], improved soil fertility [10], increased nutrient cycling [11] and soil conservation [6], improved microclimate [12], higher soil cover [13], and pest, disease, and weed control e.g., by increasing natural enemies, distancing between plants of the same species, and trapping or outcompeting harmful agents [14,15]. Disadvantages of agroforestry include competition between trees and crops for water [16], nutrients [17,18], and light [19], and increased pest and disease pressure if one component tree or crop hosts organisms can cause damage to another component crop [[20], [21], [22]]. Hence, proper design and management are necessary to ensure the sustainability of agroforestry.

Incident light to the crop canopy is one of the most important natural resources that is modified in agroforestry compared with sole cropping [23,24]. Much research has been carried out in agroforestry systems to gain a better understanding of light distribution and use by trees and understory plants. Tree canopies can intercept 10–90 % of incident light [25] The remaining light is reflected (<10 %) or available to be absorbed by crops and weeds [25]. The proportion of light intercepted by tree and crop components depends on the canopy structure [26,27], the distance between trees [19], and the ability of crops to fill and utilize gaps. Biomass production by both trees and crops is correlated with light interception [28,29]. The responses to light modified by trees differ between crop species. Positive effects of reduced light intensity include increased nutrient uptake and chlorophyll content in leaves, and more favorable microclimate close to tree canopies, in some cases resulting in increased growth rate and leaf area index (LAI) of crops [22,26,30,31].

Research on light incidence and utilization in agroforestry has focused on improving system productivity by minimizing competition between components and optimizing light capture, i.e., preventing light reaching the soil surface [30,32]. Incident light to crops and crop productivity may be reduced for crops growing close to fruit tree rows [30,[33], [34], [35]]. On flat land, the sun's direction controls incident light in the system and trees/crops can be arranged in a north-south orientation to optimize light capture and growth [36]. On sloping land, incident light is also affected by slope gradient, slope length, and slope aspect. For example, a west- or east-facing slope reduces day length. The impact increases when slope length and slope gradient increase [37]. However, it is unclear how these general factors translate into light distribution and spatial variation in growth and yield in agroforestry on sloping land. Selecting a planting arrangement to minimize slope effects is difficult, since the slope direction largely determines the planting direction (perpendicular to the slope). Enhanced knowledge of light incidence and interception on sloping land would help promote establishment of fruit trees on sloping uplands of north-west Vietnam, which cover 254,200 ha [38]. Large numbers of fruit trees are now being introduced to cropping systems that previously consisted of sole maize or coffee. Different fruit tree species differ in terms of morphology, phenology, or physiology, and companion crops can be chosen based on market opportunities or shade tolerance. This calls for knowledge and science-based recommendations on how to optimize design and management of fruit tree-crop agroforestry systems on sloping land to meet both short- and long-term sustainability and profitability goals.

The overall aim of this study was to determine how slope influences light distribution and performance of maize and coffee crops in two semi-mature fruit tree-crop agroforestry systems on land sloping to the west-southwest, to provide evidence and experimental support for system redesign and adjustment of management practices. The hypotheses tested were that 1) crops upslope of the tree rows receive and intercept greater amounts of light than that downslope; and 2) the position of the crop is more important for light interception and yield when the fruit trees have developed a large, dense canopy.

## 2 Materials and methods

### 2.1 Site descriptions

**Fig. 1:**
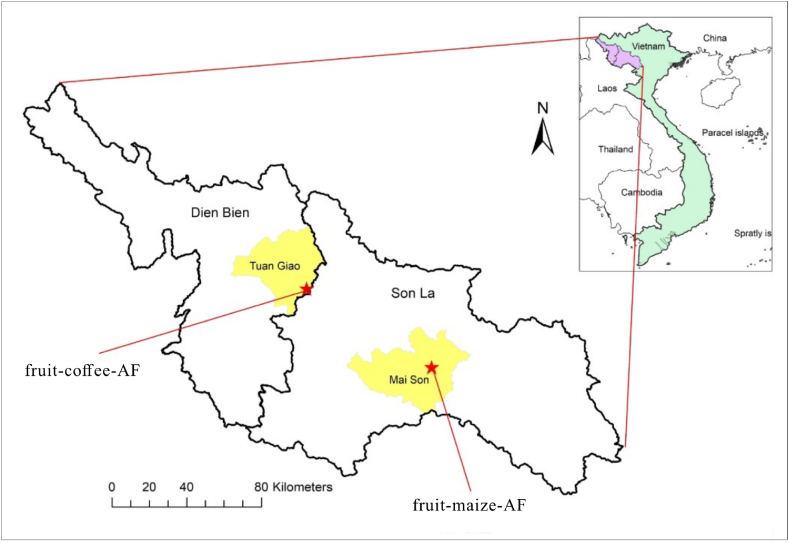
Location of the fruit tree-maize agroforestry (fruit-maize-AF) system in Mai Son district, Son La province, and fruit tree-coffee agroforestry (fruit-coffee-AF) system in Tuan Giao District, Dien Bien province.

The research was carried out in two fruit tree-crop agroforestry experiments established by the AFLi project [39] in 2017 [40]. The experiments comprised of fruit tree-maize agroforestry (fruit-maize-AF) in Mai Son district, Son La province (21.10°N, 104.06°E; 566 masl) and fruit tree-coffee agroforestry (fruit-coffee-AF) in Tuan Giao district, Dien Bien province (21.33°N, 103.30°E; 1104 masl) (Fig. 1). Both sites are characterized by a subhumid tropical climate with mean annual temperature of 21.5 °C and 18.6 °C in Mai Son and Tuan Giao, respectively. The sites have a rainy season from May to October and a dry season from November to April. Annual rainfall during the period 1989–2022 was on average 1380 mm in Mai Son and 1680 mm in Tuan Giao, mostly falling from May to August. Sole cropping was the typical practice at both sites before the establishment of agroforestry, with farmers mainly planting annual crops such as upland rice and maize in Mai Son and upland rice, maize, and coffee in Tuan Giao.

The fruit-maize-AF system has 15–26° slope (mean 21°), while that in the fruit-coffee-AF system is 24–34° (mean 29°). These ranges are representative of sloping lands in Northwest Vietnam. Both fields face west-southwest. The soils in both experiments are classified as Acrisols and have 1.8 % (Mai Son) and 2.0 % (Tuan Giao) soil organic carbon (SOC) in the Ap-horizon. The soil texture varies with depth, with clay content of 18 % (Mai Son) and 17 % (Tuan Giao) % in the Ap-horizons increasing to 42 and 30 %, respectively, in the B2 horizon (around 45–55 cm depth), and decreasing to 25 and 22 %, respectively in the BC horizon. Nutrient concentrations (especially K and P) are low at both sites and soil pH (H_2_O) is low in fruit-maize-AF (5.5) and very low (4.0) in fruit-coffee-AF. Detailed information about the soil characteristics of both sites is given by Do et al. [40].

### 2.2 Field experiments, study design, and management

#### 2.2.1 Field experiments

**Fig. 2:**
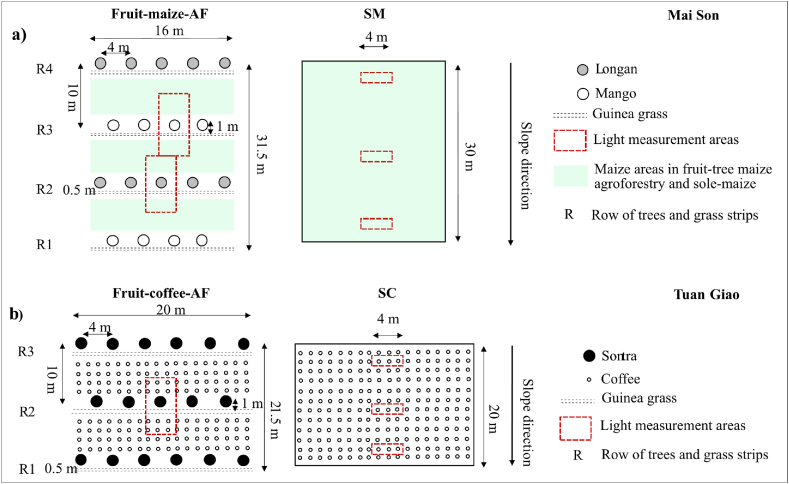
Field experiment design and data collection areas at (a) Mai Son: fruit tree-maize agroforestry (fruit-maize-AF) and sole-maize (SM) treatments and (b) Tuan Giao: fruit tree-coffee agroforestry (fruit-coffee-AF) and sole-coffee (SC) treatments. Adjusted from Do et al. [40].

The field experiments had a randomized complete block design with four replicates and two treatments: agroforestry and sole cropping (Fig. 2). In fruit-maize-AF, longan (*Dimocarpus longan* Lour. ‘PHM-99-1-1’) and mango (*Mangifera indica* L. ‘GL4’) were intercropped with maize (*Zea mays* L. ‘PAC999Super’) and guinea grass (*Panicum maximum* Jacq. ‘Mombasa’). All trees and crops were planted as single-species rows along the contour lines considering both environmental and economic aspects, and the farmers’ management techniques. Also following the dominating farmer management, the trees were free-standing and pruned as described in section 2.2.3. The distance between two rows of the same fruit species was 20 m and the distance within rows was 4 m (125 trees/ha). The longan and mango fruit species were planted in alternate rows, so that the distance between two tree rows was 10 m (i.e. in total 250 trees/ha). Double grass strips were planted at the downslope side of the tree rows, with a distance to the tree row of 1 m and a distance between two grass strips of 0.5 m. In 2022 (season six of the agroforestry system), maize was sown with 0.7 m between rows and 0.3 m within rows. The closest maize row upslope of the trees was planted 1.2 m from the tree trunks, while on the downslope it was planted 1.25 m from the center of the grass strips. Due to the increased tree canopies, maize was not sown in the fruit trees rows as done during earlier stages of the experiment [40]. Fruit trees and grass strips accounted for approximately 30 % of the land in the fruit-maize-AF system in 2022. In the sole-maize treatment, maize was sown as in the agroforestry system, but on 100 % of the land, giving a density of 71,000 plants/ha.

In fruit-coffee-AF, sontra (*Docynia indica* (Wall.) Decne.) was intercropped with coffee (*Coffea arabica* L. ‘Catimor’) and guinea grass (*Panicum maximum* Jacq. ‘Mombasa’), all planted along the contour line (Fig. 2) similar to the fruit-maize-AF. The distance between two sontra rows was 10 m and the within-row distance was 4 m (250 trees/ha). Double grass strips were planted as in fruit-maize-AF. Between two sontra rows, four coffee rows were planted with 2 m between rows and 1.4 m within rows. The nearest coffee row on the upslope of the trees was 1.5 m from the sontra row, while on the downslope it was 1.25 m from the center of the grass strip. In the sole-coffee (SC) system, coffee was planted with the same distance between and within coffee rows as in the agroforestry system.

#### 2.2.2 Study design

**Fig. 3:**
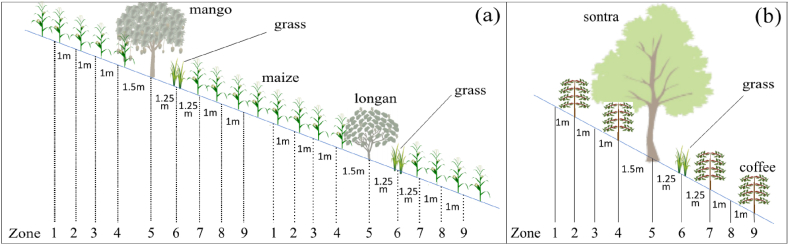
Center of zones in longan-maize-grass (longan-AF) and mango-maize-grass (mango-AF) sub-systems in (a) the fruit tree-maize agroforestry (fruit-maize-AF) and (b) fruit tree-coffee agroforestry (fruit-coffee-AF) systems. The general shape and size of the tree canopies is indicated by the size of the icons. Icon sources: https://depositphotos.com/vectors/tree.html.

In fruit-maize-AF, the agroforestry plots were divided into two AF sub-treatments with longan-maize-grass (longan-AF) and mango-maize-grass (mango-AF) sequences, respectively. To test the hypothesis on light distribution, nine zones were identified along the slope of all agroforestry plots in both experiments: zones 1 to 4 on the upslope side and zones 6 (grass strips) to 9 on the downslope side of tree row (zone 5). The width of each crop zone (1–4 and 7–9) was 1 m, as the center of two neighboring crop zones was 1 m apart. The center of zone 4 was 1.5 m from the tree trunks. The center of zone 6 and zone 7 were located 1.25 m from the tree trunks and zone 6 center, respectively (Fig. 3).

#### 2.2.3 Management of experimental treatments

Maize was sown on May 14, 2022, following application of NPK (5:10:3) basal fertilizer. The maize was weeded and then top-dressed with urea and potassium at 6–7 leaves and silking stages. The total amount of nutrients applied to maize in the agroforestry sub-treatments was 192 kg N, 18 kg P, 63 kg K, and 40 kg S ha^−1^, which was 30 % lower than in sole-maize, treatment reflecting the smaller maize area (details in Tables S1 and S3 in Supplementary Data). Fruit trees were fertilized three times (in March, June, September), with a total amount of 0.32 kg N, 0.091 kg P, 0.207 kg K, and 0.035 kg S tree^−1^. In the 2022 season, fall armyworm (*Spodoptera frugiperda* (J.E. Smith)) was controlled by emmabectin benzoate active ingredient twice in June. Fipronil active ingredient was applied once, in September, to protect the fruit tree buds against young twig borer (*Niphonoclea albata*) and leaf-eating insects (*Adoretus* sp.). In fruit-coffee-AF, sontra and coffee were fertilized three times (in March, June, September), with a total of 200 kg N, 61.5 kg P, 204.6 kg K ha^−1^ and 0.18 kg N, 0.135 kg P, 0.075 kg K tree^−1^, respectively (details in Tables S2 and S4). Coffee shrubs were sprayed with acetamiprid and chlorpyrifos ethyl active ingredients twice in March to control coffee scale bug (*Coccus viridis*). No fertilizer was applied to the grass in any of the experiments.

At the time of the light measurements, the trees were five years old and were bearing fruits. The mango and longan trees were pruned three times during the growing season and once after harvest according to common practice. The major pruning was in winter (November-early December) when approximately 20 % of the canopy was removed, while some gentle pruning to manage twig density was done during the summer (May–June) and autumn (August–September). After harvesting fruits in June (mango) or in September (longan), farmers cut all dead branches and fruited twigs. The sontra trees in fruit-coffee-AF were only pruned during the winter, and the pruning then restricted to removing lower branches and twigs to avoid them weighing down on the coffee shrubs. The coffee shrubs were pruned regularly in spring and summer by the host farmers, as they wanted to maintain a height of 1.6–1.7 m.

Weeding was performed several times in both experiments. In fruit-maize-AF, farmers hand-hoed before the sowing of maize as part of land preparation for maize cultivation, and again at the 6–7 leaf and silking stages, immediately before fertilization. Farmers weeded again, after harvest, in December, using a strimmer. All weed and maize residues were left in the field. In fruit-coffee-AF, farmers weeded the plots three times, by strimmer in March and September and by glufosinate ammonium in June. Details of field management in previous years (2017–2021) are given by Do et al. [40].

### 2.3 Data collection

In order to explore the distribution of light in the agroforestry systems, we collected weather data and measured incident light at crop level and light interception by the crop layer once every third month, starting in March and finishing in December. Additional measurements of light were carried out in fruit-maize-AF at the maize growing stages of 3–4, 6–7, 10–11 leaves, and silking. The growth and yield performance of crops and trees were monitored to test the relationship between crops and light distribution.

#### 2.3.1 Total incident light, rainfall, and temperature at the study sites

A mini-weather station (ATMOS 41, METER Group, Inc.) was installed in the middle of fruit-maize-AF to determine incident light, rainfall, and air temperature. Incident light to fruit-coffee-AF was estimated from daily temperature [41,42], using temperature data from a nearby weather station. Rainfall was recorded manually in another experiment approximately 2 km from the fruit-coffee-AF experiment.

**Fig. 4:**
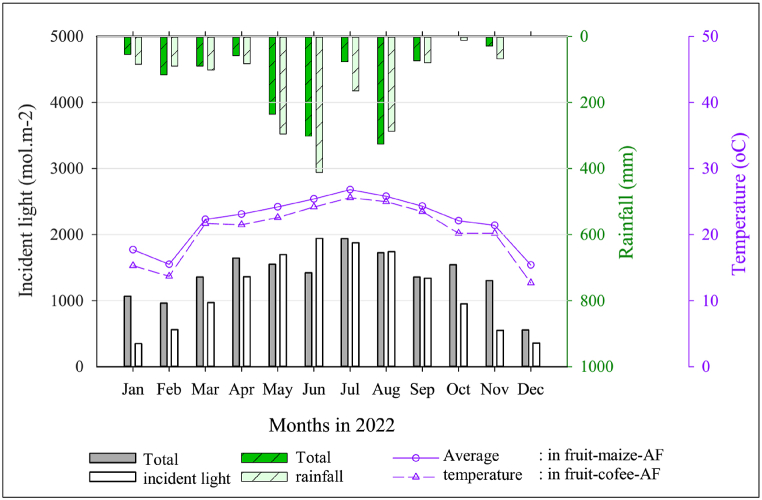
Monthly total incident light, rainfall, and average temperature in the fruit tree-maize-agroforestry (fruit-maize-AF) and fruit tree-coffee-agroforestry (fruit-coffee-AF) systems in 2022.

Total incident light to fruit-maize-AF and fruit-coffee-AF was approximately 16,300 and 13,300 mol m^−2^, respectively. Light intensity showed a peak in July–August in fruit-maize-AF, and in June–July in fruit-coffee-AF (Fig. 4). Total annual rainfall was 1365 mm in fruit-maize-AF, while fruit-coffee-AF received 1676 mm. Rain was concentrated to four months (May–August), which accounted for approximately 70 % of the total annual amount. Mean monthly temperature was above 20 °C except in December–February, and higher in fruit-maize-AF than in fruit-coffee-AF.

#### 2.3.2 Light distribution

We combined an indirect method (Hemiview) and a direct method (SunScan) to assess light distribution. Hemiview can be used to assess the incident light above the tree canopy for the measurement day using field configurations but cannot be used at the soil surface. On the other hand, SunScan can measure light at specific times and at the soil surface, but data collection is limited by time resources (labor) and ability to work above the tree canopy. A strong correlation (R^2^ = 0.949) was found by Hale [43] when comparing measurements from the two methods. Both methods have been used in combination by other researchers to investigate light distribution, e.g. Dong et al. [44], or to validate other methods, e.g. Zhao et al. [45].

A Canon EOS 60D digital single-lens reflex camera and fish-eye lens (Sigma EX DC 4.5 mm) were connected to a HemiView frame and a monopod and used to take hemispherical images at 1.7 m height (crop level, at the approximate maximum height of the maize and coffee canopies). All images were processed by HemiView canopy analysis software to calculate the fraction of incident light reaching the crop level (F_*incident*_ _*to*_ _*crop*_). Field criteria, including longitude, latitude, altitude, slope, and measurement date, were included in the software to configure the analysis model. The threshold value was adjusted by the analyzer to avoid noise from clouds. The time in the software was set to the time of image capture. The magnetic declination value was calculated using a tool made by NOAA [46]. In Skymap, the azimuth divisions were set to 8 (45-degree divisions) and the zenith divisions to 18 (5-degree divisions), the settings typically recommended for analysis of hemispherical photographs [47]. For configuring the intercepting surface, the azimuth was set as 0, since the camera was kept at constant orientation with the support of a compass attached to the HemiView frame. The zenith value was the mean of the field slope gradient. The active side was set as single, meaning light was assumed to be intercepted by the upper part of the leaves. All images were cropped to remove interference from the camera lens. The fraction of incident light to the two agroforestry treatments was assumed to be equal to that reaching the sole-crop treatments.

Photosynthetically active radiation was measured using the SunScan canopy analysis system (SS1-COM, Delta-T), which includes a SunScan probe (SS1), a Sunshine sensor (BF5), and a handheld computer (PDA). The SS1 connects to the PDA and consists of 64 PAR sensors embedded evenly in a 1-m probe. The BF5 was placed in the middle of sole-crop plots at 1.7 m height and connected to the SS1 by a cable to provide the reference for SS1 measurements in the AF plots. In each crop zone of the agroforestry sub-systems and sole-maize system, five measurements were made 1 m apart within a maize row and five in a line half-way between two maize rows, both being closest to the middle of the zone. These measurements were at two levels, i.e., at crop level (1.7 m height, level I_1_) and at the soil surface (level I_2_). In zones 5 and 6, measurements were taken at five points along the center of the rows (tree rows and between two grass strips, respectively). In fruit-coffee-AF, five points, 1m apart, were measured at two levels in the centerline of each zone.

At each measurement point, the soil surface was measured first and then the SS1 was quickly moved to 1.7 m height, to minimize the effect of weather conditions. Measurements were made between 10.00 and 14.00 h on a sunny day with a clear sky, according to recommendations [48], as the sun attributes would be most stable and the effect of environmental conditions such as cloud, moisture, or haze on the light fraction minimized. The SS1 was placed horizontally along the contour line. To further minimize the effect of environmental conditions, fractions (F) were calculated. The fraction of light intercepted by the crop layer (F_crop_ _interception_, layer between 1.7 m height and soil surface) was calculated by Eq. (1) based on incident light at crop level (F_incident_ _to_ _crop_), I_1_, and I_2_:

(1) *F*_*crop*__*interception*_ = *F*_*incident*__*to*__*crop*_ × *(I*_*1*_*- I*_*2*_*)/I*_*1*_

In the crop zones (1–4, 7–9), the crop layer was mainly crops, while in the fruit tree (5) and grass strips (6) the crop layer consisted of grass, weeds, and/or a part of the tree crown.

The light reaching the soil surface (*F*_*light*_ _*reaching*_ _*soil*_) was calculated as the difference between F_incident_ _to_ _crop_ and F_crop_ _interception_) and shown in Eq. (2):

(2) *F*_*light*__*reaching*__*soil*_ = *F*_*incident*__*to*__*crop*_*- F*_*crop*__*interception*_

The difference in latitude between the two sites is very small, and according to Miller [37] would not substantially influence the solar zenith and azimuth, which determine the energy of incident light. Besides, since the slope aspect at both sites is close to 240° from North, we assumed that they had similar sunlight regime during the year. Seasonal average *F*_*light*_ _*reaching*_ _*soil*_, *F*_*incident to crop*_, and *F*_*crop interception*_ were computed as the average of measurements during the cropping season in both fruit-maize-AF and fruit-coffee-AF.

#### 2.3.3 Performance of trees and crops

Tree trunk diameter (D10) at 10 cm height above the ground (due to grafting and pruning practices) [49], canopy width, and tree height were measured quarterly, in March, June, September, and December, using caliper, bamboo poles, and tape measure. Similar measurements were taken on the coffee shrubs, although stem diameter (D15) was measured at 15 cm height above ground [50].

Five maize plants in each maize zone were selected randomly to measure height, SPAD, and leaf area at the 3–4, 6–7, and 10–11 leaf stages, and silking. A SPAD reader (SPAD 502 Plus Chlorophyll Meter, Spectrum Technology Inc.) was used to assess the chlorophyll concentration. Three SPAD readings were taken, at 25 %, 50 %, and 75 % along the leaf and near the leaf's midrib of the 3rd, 6th and 10th fully expanded maize leaf and the ear position leaf at the respective growing stages, and the average was calculated. In addition, the length and width of all living leaves on these maize plants were measured to calculate the leaf area of each plant (Eq. (3)):

(3) *LA* = *(L*_*1*_ × *W*_*1*_ + *L*_*2*_ × *W*_*2*_ + *…* + *L*_*i*_ × *W*_*i*_*)* × *0.73* (cm^2^)

where L_i_ and W_i_ are the length and width (cm), respectively, of the living leaf number I and 0.73 is a shape constant of the maize leaf [51].

Maize total aboveground biomass in the field was determined by cutting and weighing all maize plants in a sample area of 3.5 m^2^ in each zone. Five random plants per zone were sampled and separated into stalks, leaves, cob, grain, and ear husk. All plant samples were weighed before and after sun-drying. Coffee cherries (the fruit that contains the coffee bean) were harvested four times from September to December. Farmers picked the ripe cherries on the first three occasions, while they picked all remaining cherries on the fourth occasion. The crop yield in each zone was computed based on crop area to discuss the links with light distribution. The system productivity for the years 2017–2021 was assessed by Do et al. [49].

### 2.4 Statistical analysis

All data analysis was done using R software (version 4.1.1) and R-studio software (version 2022.12.0.353), applying a statistical significance level of p < 0.05. The statistical significance of explanatory variables was performed using ANOVA type II Wald F tests with Kenward-Roger degree of freedom to evaluate the difference between the two agroforestry sub-treatments and the sole-maize, and between zones in the agroforestry sub-treatments. In some cases, Box-Cox or square root transformation was used to fulfill the assumption of normal distribution of the residuals. Ad-hoc pairwise analysis with the Tukey adjustment method was used to compare differences between categories. A simple linear regression model with F-test was used to test the relationship between average incident light to the crop and light interception by the crop layer on the one hand, and crop growth and yield on the other hand. Another regression tested the relationship between incident light to the crop and plot slope within each experiment.

## 3 Results

### 3.1 Tree performance

In the fruit-maize-AF system, mango trees grew faster than longan. Mean mango tree height and canopy width were 3.3 and 2.9 m, respectively, almost 1.5 times greater than those of longan. The mango canopy often reached over zone 4 and zone 6, while the longan canopy rarely did. Mean stem diameter of mango (12.3 cm) exceeded that of longan (7.3 cm) (Table S5). In the fruit-coffee-AF system, the sontra trees were over 7 m tall in December 2022 and their canopy was approximately 6 m wide. The tree canopy thus reached over the nearest coffee rows in zones 4 and 7.

### 3.2 Effect of slope on incident light

On each measurement occasion, fruit-maize-AF received a significantly higher fraction of light than fruit-coffee-AF, which had a steeper slope. Incident light to fruit-maize-AF was relatively similar during the year (Table S6), while incident light to fruit-coffee-AF was variable, being higher in quarter 2 than quarters 3 and 4. Within each experiment, the regression analysis between incident light and slope range showed no significant relationship (p > 0.05).

### 3.3 Light distribution in the fruit tree-maize agroforestry system

#### 3.3.1 Incident light at maize level

**Fig. 5:**
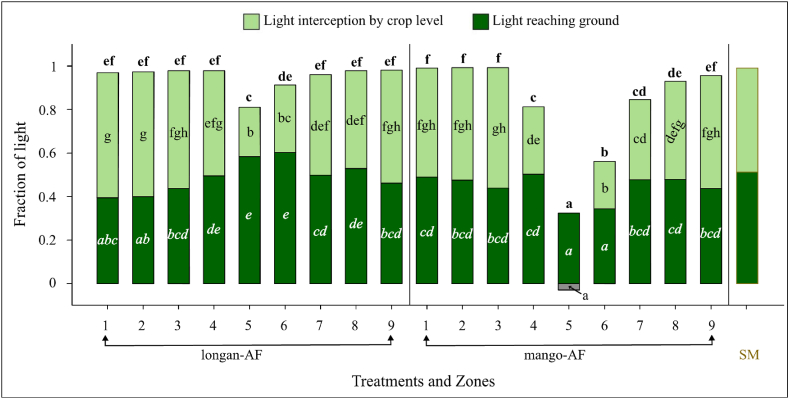
Light distribution and interception in different zones in the longan-maize (longan-AF) and mango-maize (mango-AF) agroforestry sub-treatments and in sole-maize (SM), expressed as average over the maize season. Different bold, regular, and italic letters (a, b, …) indicate significant (p < 0.05) differences between zones within agroforestry sub-treatments in terms of incident light to maize level, light interception by maize layer, and light reaching the soil surface, respectively. The grey histogram indicates that the amount of light reaching the soil surface exceeded that reaching the 1.7 m level of the mango canopy.

Sole-maize received the highest fraction of incident light at maize level (p < 0.01), followed by longan-AF and then mango-AF. The fruit tree canopy five years after establishment of the agroforestry system intercepted on average 0.05 and 0.20 fraction of total incident light in longan-AF and mango-AF, respectively. Within each sub-treatment, tree light interception varied between zones. Tree canopy had a stronger effect on incident light reaching the maize downslope than upslope. Concerning the main effect of zones, zones 4 and 6 received a lower light fraction than the remaining zones. The effect of mango on incident light at maize level was significantly greater than that of longan in zones 4, 7, and 8 (Fig. 5). The influence of both fruit trees decreased when the distance from tree rows increased.

#### 3.3.2 Light interception by the maize layer

Light interception by the maize layer differed significantly between the growth stages over the maize season (p < 0.001). On average, the maize intercepted 0.48 fraction at 6–7 leaves, 0.63 at 10–11 leaves, 0.51 at the silking stage, and 0.20 at harvest time. The mango trees had a greater effect than longan trees on light interception by the maize layer in zones 4 and 7, but the effect decreased with increasing distance from the tree rows (Fig. 5). The negative value of light interception in zone 5 of mango-AF shows that the incident light at the 1.7 m level of the tree canopies was less than the light reaching the ground, probably due to light coming in from the sides close to the ground.

#### 3.3.3 Light reaching the soil surface

Before the pre-planting weeding for maize, the soil surface in sole-maize, longan-AF, and mango-AF plots received an average of 0.84, 0.83, and 0.80 fraction of total incident light, respectively. During the maize season, light reaching the soil surface did not differ between the (sub-)treatments (p = 0.89), but differed between maize development stages (p < 0.001) in the order: 10–11 leaves < silking < 6–7 leaf stages. The soil surface in the maize zones in both agroforestry sub-systems received more light than zones 5 and 6 during the off-maize season. A similar trend was found in mango-AF during the maize season, but zones 5 and 6 in longan-AF showed the opposite (Fig. 5).

### 3.4 Light distribution in the fruit tree-coffee agroforestry system

#### 3.4.1 Light incidence at coffee level

**Fig. 6:**
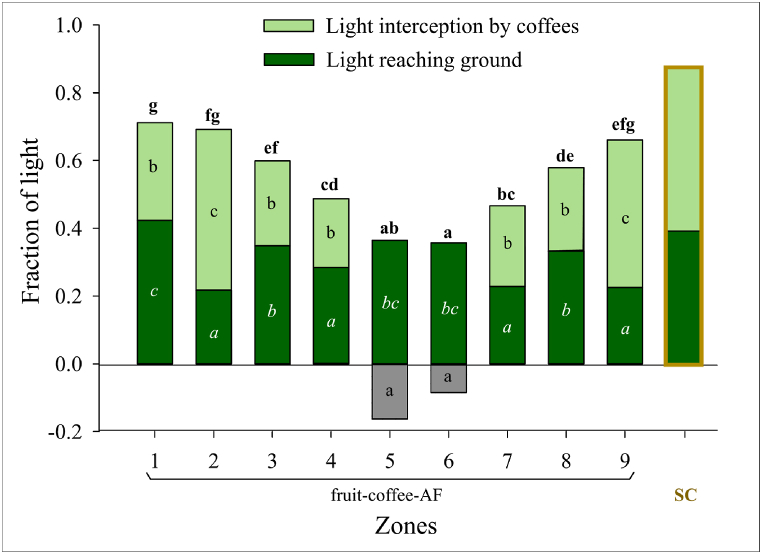
Mean annual light distribution in the fruit tree-coffee agroforestry (fruit-coffee-AF) and sole-coffee (SC) systems. Different bold, regular, and italic letters (a, b, …) indicate significant differences (p < 0.05) between zones in fruit-coffee-AF in terms of incident light at coffee level, light interception by coffee layer, and light reaching soil surface, respectively. The grey histograms indicate that the amount of light reaching the soil surface exceeded that at the 1.7 m level in the fruit tree rows and grass strips.

Sole-coffee shrubs received a larger fraction of the incident light than coffee in the agroforestry treatment across all four measuring occasions of 2022 (Fig. 6). Incident light to the coffee increased with increasing distance from the sontra row both upslope and downslope (p < 0.001). Zones on the downslope of sontra tree were slightly more impacted than upslope zones on the same distance from the tree row.

Within sole-coffee, the difference in fraction of intercepted light between measuring occasions was small (0.84–0.92). However, in the agroforestry treatment it was lowest in quarter 3 (0.49 as averaged across all zones) and highest in quarter 4 (0.61), reflecting sontra growth and leaf-drop.

#### 3.4.2 Light interception by the coffee layer

Light interception by the crop layer was higher in sole-coffee than in the agroforestry treatment (p < 0.05). It was significantly lower in quarter 3 than in quarters 1 and 4 (p = 0.003). In fruit-coffee-AF, the coffee layer intercepted from 0.20 to 0.50 fraction of the total incident light, which declined rapidly with shorter distance to the tree row. Grass strips and tree rows received lower light intensity at the 1.7 m (crop) level than at the soil surface (Fig. 6).

#### 3.4.3 Light reaching the soil surface

The soil surface in sole-coffee received significantly greater amount of light than that in agroforestry (p = 0.003). In the agroforestry treatment, light reaching the soil surface was lower within the coffee rows (zones 2, 4, 7, and 9) than between rows (zones 1, 3, and 8) (p < 0.001). Significantly more light reached the soil surface in the tree row than in all other zones except for grass strips and the middle alley (zone 1) (Fig. 6).

### 3.5 Crop growth and yield

#### 3.5.1 Maize growth and yield in the fruit tree-maize agroforestry system

Maize showed best performance in sole-maize, followed by the plot averages of longan-AF and mango-AF sub-treatments. The maize in sole-maize was significantly taller than in the mango-AF (Fig. S1). The leaf area was also larger in sole-maize than in mango-AF at the 3–4, 6–7 and 10–11 leaf stages, but not at the silking stage. Maize height and leaf area in the longan-AF sub-treatment was intermediate and not significantly different from the others. The leaf SPAD value increased in all three (sub-)treatments from the 3 to 4 leaf stage to the silking stage, but there was no significant effect between the treatments.

**Fig. 7:**
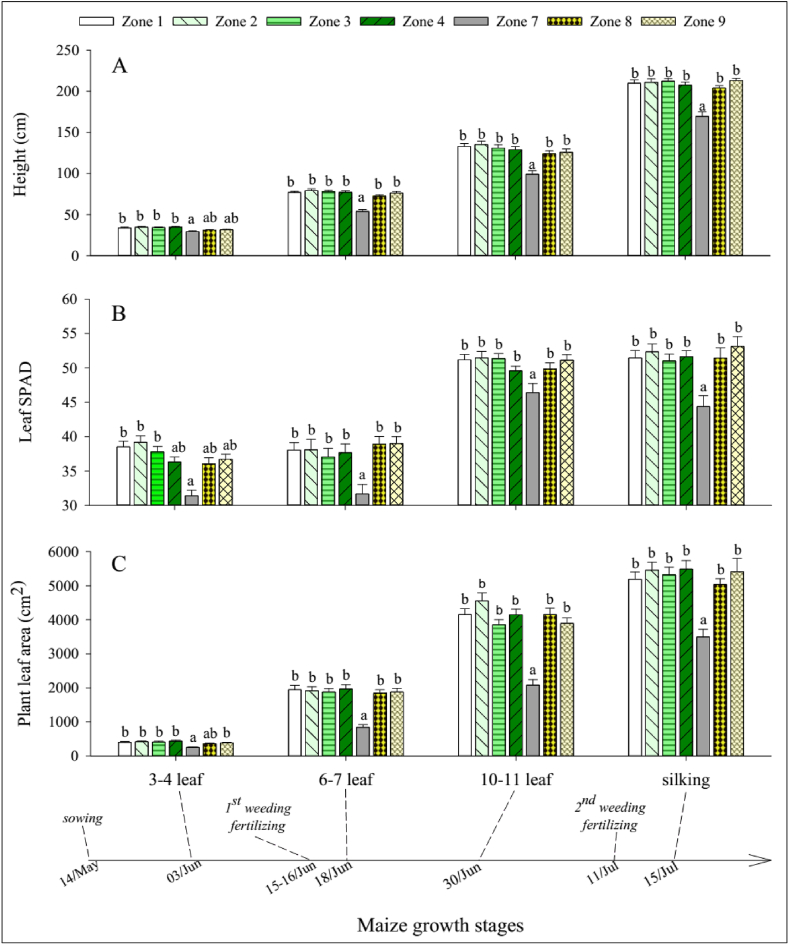
(A) Maize height, (B) leaf SPAD value, and (C) leaf area in different maize zones and development stages across agroforestry sub-treatments. Different letters (a, b) indicate significant differences between zones within the respective development stage (p < 0.05).

The effects of competition were mainly found in the nearest maize zone on the downslope side of tree rows. Maize height (Fig. 7A), leaf SPAD values (Fig. 7B), and leaf area (Fig. 7C) in zone 7 were significantly lower than in other zones (p < 0.001) during all maize development stages in both longan-AF and mango-AF.

**Fig. 8:**
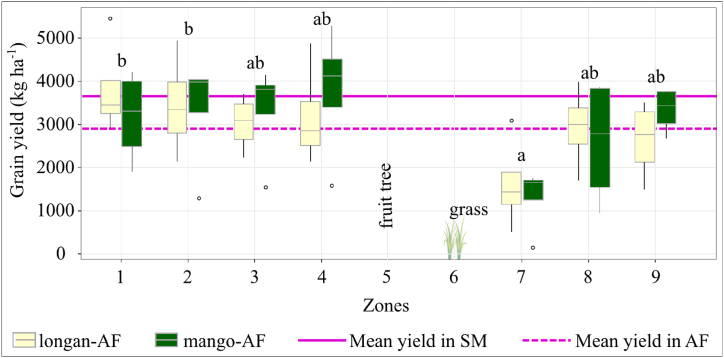
Grain yield in maize zones in the longan-maize-grass (longan-AF) and mango-maize-grass (mango-AF) agroforestry sub-treatments. The main effect of zone was significant (p < 0.001). Different letters (a, b) indicate significant differences between maize zones (p < 0.05). The error bars show 95 % confidence intervals.

Mean maize grain yield and aboveground biomass did not differ significantly between the sole-maize and the agroforestry sub-systems. Zone 7 had significantly lower yield and biomass in both agroforestry sub-systems (Fig. 8, Fig. S2). There was a tendency for grain yield and aboveground biomass to decrease with shorter distance from maize zones to the tree row on the downslope side of the trees.

#### 3.5.2 Coffee growth and yield in the fruit tree-coffee agroforestry system

There were no significant differences between the sole-coffee and agroforestry treatments in coffee height and canopy width (p > 0.05). Mean coffee stem diameter tended to be slightly larger in sole-coffee than in agroforestry, with the difference being significant in December (Fig. S3). The SPAD values decreased gradually over the year and were lower in agroforestry than in sole-coffee in March.

**Fig. 9:**
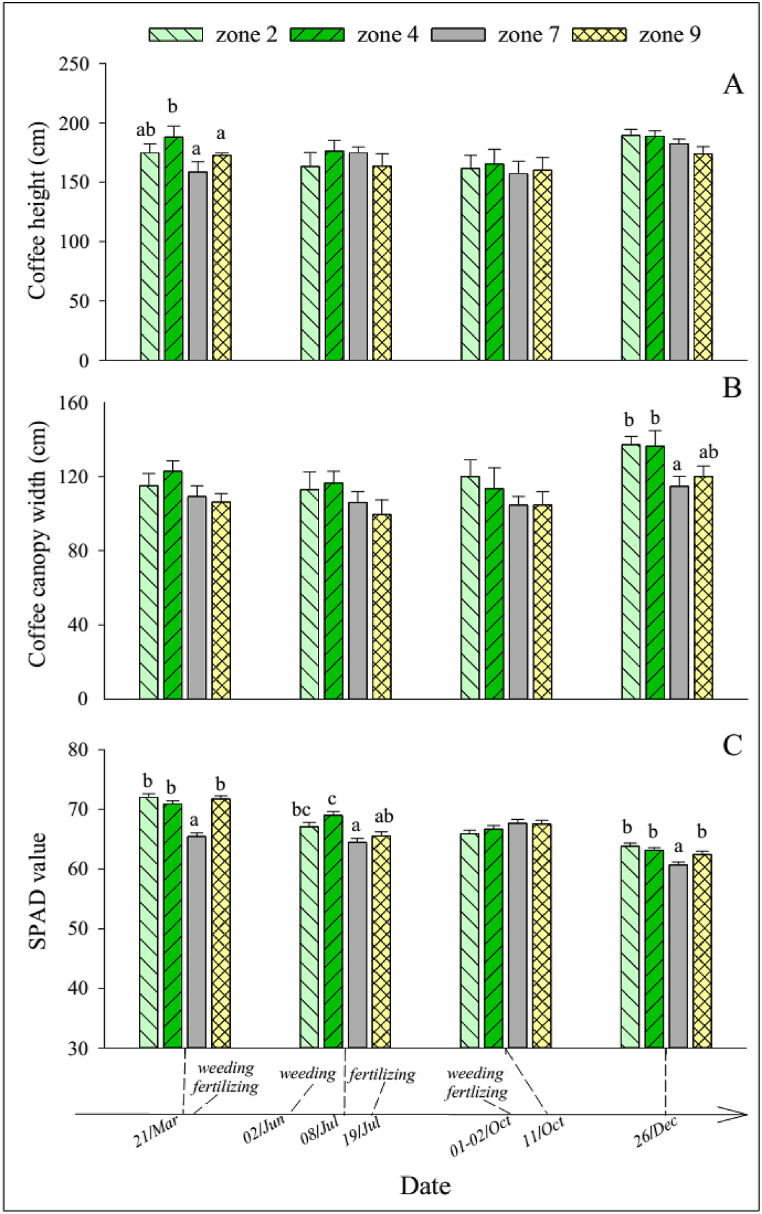
(A) Coffee shrub height, (B) canopy width, and (C) leaf SPAD value in different zones of the sontra-coffee-grass (fruit-coffee-AF) agroforestry system in 2022. Error bars show 95 % confidence interval. Different letters (a–b) indicate significant differences within the respective development stage (p < 0.05).

Coffee height in zone 7 in the agroforestry treatment was significantly lower than in the other coffee zones in March (Fig. 9A). Coffee canopy width in zones 7 and 9 tended to be slightly smaller than in zones 2 and 4, but a significant difference was only found in December (Fig. 9B). Coffee stem diameter was not significantly different between the zones. Leaf SPAD values were lowest in zone 7 in March, July, and December (Fig. 9C).

**Fig. 10:**
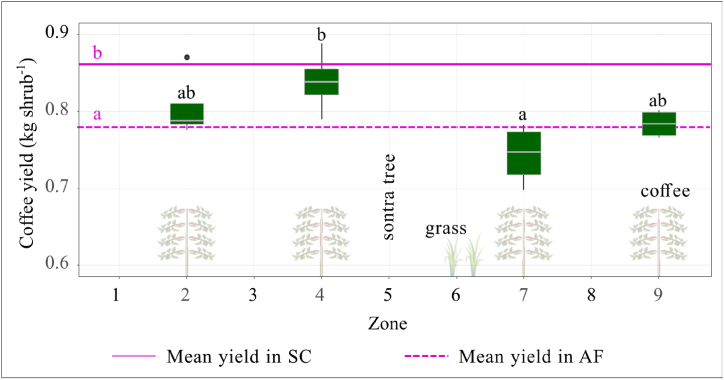
Fresh coffee cherry yield in agroforestry (AF) and sole coffee (SC) plots in the sontra-coffee-grass (fruit-coffee-AF) system. Error bars show 95 % confidence interval. Different black and purple letters (a and b) indicate significant differences between zones and treatments, respectively (p = 0.05). (For interpretation of the references to colour in this figure legend, the reader is referred to the Web version of this article.)

Sole-coffee had significantly higher fresh cherry yield than the average of the coffee zones in agroforestry (Fig. 10). In the agroforestry treatment, coffee on the downslope of the tree row tended to have lower yield than that on the upslope, but the difference was only significant between zone 4 and zone 7.

### 3.6 Correlation between light and crop performance

In fruit-maize-AF, there was no significant linear relationship between incident light at maize level and maize performance (p > 0.05). In contrast, light interception by the maize layer was positively correlated with all maize variables, including height, SPAD value, leaf area, grain yield, and total aboveground biomass (Fig. S4). As in fruit-maize-AF, there was no correlation between incident light at coffee level, and coffee performance and yield in fruit-coffee-AF. Light interception by the coffee layer showed no significant correlation with coffee growth variables and fresh cherry yield (p > 0.05).

## 4 Discussion

### 4.1 Light distribution in the agroforestry system on sloping land

Approximately 0.99 and 0.87 fraction of total incident light reached to the crop level in sole-maize and sole-coffee, respectively. The difference was probably caused by the steeper slope in the fruit-coffee-AF experiment, which delayed the sunrise and shortened the day length [37]. Contributing factors could also have been the longer upslope hill above the fruit-coffee-AF experiment and taller trees around the experiment. This suggests that field experiments under these conditions need larger plot areas and appropriate buffer areas between treatment plots, and that all plots should be established along the same contour line to avoid the influence of upper treatment plots on downslope plots.

The seasonal average amount of fraction of incident light at crop level in different zones varied from 0.40 to 0.99 in fruit-maize-AF and from 0.40 to 0.73 in fruit-coffee-AF. The amount of incident light was lower closer to the tree rows than farther away, in agreement with findings by Nicodemo et al. [52] and Abbasi Surki et al. [19]. Fruit tree size (such as height, canopy width, and canopy structure) plays an important role in incident light at crop level [8]. Therefore, the light distribution in an agroforestry system varies depending on the system design, including the choice of tree/crop components and their allocation in the field. The experimental design in the study plots was informed by both economic and environmental targets, and therefore used a distance of 10 m between two fruit tree rows, which is suggested as optimal by the International Center for Research in Agroforestry [53]. On flat land, the lower risk of soil erosion allows farmers to use a greater distance between tree rows [54] and therefore the effect of trees on incident light can be kept lower than in agroforestry on sloping land.

The incident light at crop level was less affected upslope of the tree rows than downslope, which to our knowledge is a novel finding. The slope reduced the altitude of the tree canopy relative to the crop level on the upslope side of the trees, but increased it on the downslope side. At both sites, with west-southwest facing direction, downslope crops received a smaller proportion of the sunlight than upslope crops because of more tree shading earlier in the day. This was clearer in fruit-maize-AF, where the fruit trees were smaller with regular canopy management, than in fruit-coffee-AF, where the tree canopies were not managed and therefore larger, and shading was more similar throughout the day.

Within the fruit-maize-AF system, the soil surface in tree rows and grass strips in the longan-AF sub-treatment received a greater fraction of sunlight than the crop canopy level (1.7m), especially at the 6–7 and 10–11 maize leaf stages. A similar increase in incident light between crop canopy level and soil surface was observed in the fruit-coffee-AF system. This increase in light between the two levels could be due to reflection from other system components, diffuse light, direct sunlight in mornings and evenings when sun was low [55], and the fact that there was little vegetation below the trees in zone 5.

### 4.2 Crop performance in relation to light distribution under the tree canopy

Plants can adjust their organ function to adapt to changes in light availability [56]. However, their ability to adjust is weak under inhomogeneous light distribution, as in agroforestry systems, and plants experiencing inhomogeneous light can therefore be expected to be more negatively influenced than those under homogeneous low light [57]. However, shading is only a problem if light is the limiting factor for growth, and this is often not the case in agriculture [58]. Crop performance in our experiments differed between crops upslope and downslope of the tree rows. In both experiments, yields were lower on the downslope side of the tree rows than upslope. Lower yield of crops closer to the tree rows, as found on the downslope side, is in line with findings on flat land [30,33,34,59,60]. The effect of tree rows on crop performance on the downslope side was similar to their impact on incident light at crop level. This suggests that the strong effect of the trees on incident light on the downslope side was the cause of the poorer crop performance. However, it might also be caused by competition for water and nutrients [[60], [61], [62], [63]] on the downslope side of the trees where double grass strips were planted to reduce soil erosion [40]. Previous studies have shown that erosion occurs below grass strips, whereas the upslope side tends to accumulate soil and water [40,64]. On the upslope side of the tree rows, we saw no trend of declining yields closer to the tree rows. The maize and coffee rows nearest above the fruit trees even tended to perform better than those farther away from the tree rows, indicating that the better performance of crops upslope than downslope of trees was not only due to less shading but most likely also competition from grass roots downslope. The crops immediately above the tree rows might also have utilized part of the nutrients applied to the trees, and might have benefited from favorable environmental conditions that trees could provide, such as lower wind velocity, better water availability, and mitigation of extreme weather events, as summarised by Nair and Garrity [65].

Incident light was thus apparently not the limiting factor for maize and coffee in our study. In fruit-maize-AF, zone 4 received an average of 0.82 fraction of total incident light and performed similarly to the middle alley (zone 1, 2), which received approximately 0.99. Maize, an annual C4 plant, has high potential photosynthetic rates at unlimited sunlight and high temperature [66], and is therefore considered to be highly sensitive to light limitation [[67], [68], [69]], which reduces its growth and yield [[70], [71], [72]]. We observed a weak correlation between light interception by maize and maize performance, so the increased light interception by the crop was probably caused by the greater maize biomass enabled by higher availability of nutrients and/or water [73,74]. In the fruit-coffee-AF system, coffee received 0.50–0.70 and intercepted 0.20–0.50 fraction of total incident light, and apparently adapted well to the shaded conditions, corroborating findings by Soto-Pinto et al. [22] that 38–48 % shade cover produces the highest coffee yield. However, Muschler [75] found that coffee performed differently when intercropped with different tree species and at reduced distance to tree rows, due to variations in competition, compatibility, weeds suppression, and disease control [76]. The reaction to shading may also vary depending on coffee cultivar [77].

### 4.3 System modification to optimize light capture

Management practices play a crucial role in modifying light distribution in agroforestry. Farmers usually apply cultivation techniques to accomplish particular goals, especially higher productivity and quality. One of the most common techniques is pruning woody trees and shrubs. In fruit-maize-AF, farmers carried out pruning/thinning 3–4 times a year to manage fruit tree shape and density and stimulate growth of new shoots with high-quality flower buds. Pruning and thinning also reduce the competition by the tree component in agroforestry [27,30,78,79]. In fruit-coffee-AF, farmers cut the lower branches/twigs of sontra trees once during the winter season to prevent them from collapsing onto the coffee shrubs and to facilitate other management practices such as pruning coffee shrubs, cutting grass, fertilizing, and weeding. More elaborate pruning and thinning are not usually applied to sontra trees, because farmers do not anticipate a sufficient increase in payment to offset the labor cost for pruning and they are also often unwilling to use a new practice, such as pruning of sontra, until a clear benefit has been demonstrated. Therefore, we followed the general practice and only did the minimum pruning required to facilitate other activities in the experiment. To promote quality-enhancing management strategies, there is a need to develop the market to increase price and income from quality fruit. There is a general lack of research on how sontra reacts to pruning and other management [80].

Competition can also be regulated by tree row arrangements. On flat land, north-south tree lines are recommended at high and medium latitudes to achieve homogeneous light for crops in the alley [81] while at low latitudes the direction of the rows is less important [82]. The possibility to decide row-orientation on sloping land is often limited because planting is preferably done along contours to reduce erosion and to facilitate management. However, the spacing within and between tree rows can be optimized if sufficient knowledge about resource partitioning and resource use efficiency are available [8]. To compensate for the effect of soil cultivation on soil erosion, other soil conservation measures can be integrated into the system, such as artificial terraces [83], vegetative sediment traps [84], or legume strips [85]. Changing the planting pattern such that trees and crops are assigned to the most suitable fields at landscape level would be an option, but would require consensus among farmers, and the possibilities for farmers to adapt crops to different fields are limited due to the small size of each farm.

The amount of light intercepted by the system can be increased through agroforestry practices and maintaining living vegetative cover, while ensuring that the different components are managed to achieve appropriate interactions. System modifications to achieve special goals can be made by selecting crops with suitable levels of competitive ability in time and space, managing tree and crop density, scheduling planting or sowing, fertilizing, managing weeds, pests and diseases, irrigating, and pruning tree canopy [86,87]. If fruit production is the priority, adjustments should focus on increasing tree density and applying tree management that enhances fruit yield and quality. On the other hand, if farmers prioritize understory crops or pastures giving immediate returns, managing the amount and pattern of light transmittance is more crucial [29]. Coltri et al. [88] emphasized overstory management as important to create suitable conditions for understory crops and reduce possible climate stressors. Such management depends on the architecture and seasonal growth pattern of the trees, especially for deciduous species with distinct bud bursts. Mango has a denser canopy than longan, but both are evergreen species [89,90] and maintain relatively stable light to lower levels. On the other hand, sontra is a deciduous species [80,91] with larger fluctuation in shading over the year. Our results suggest that in fruit-maize agroforestry, more severe pruning of mango trees should be implemented to reduce the shading effect and maintain a more uniform light regime. In fruit-coffee agroforestry, different pruning strategies should be tested and focused on improving sontra yield and quality.

Coffee maintains a perennial canopy, but maize has a growth cycle of only about four months at the study sites [92], leading to poor utilization of light during much of the year. Fruit-maize-AF systems should be modified to increase and prolong the vegetative cover by introducing long-life cycle crops, intercropping with e.g., leguminous species, crop rotation, or relay-cropping. For long-life cycle crops, cassava can be considered. This species takes approximately one year to complete its life cycle in the study area, and maintains living vegetative cover over the dry season [93,94]. In fact, at the fruit-maize-AF site, some farmers have replaced maize with cassava. Others have planted sugar cane, but its high competitiveness raises questions about trade-offs in light, nutrient, and water use. The long dry season during winter and subsequent water restriction is the greatest challenge for farmers who want to add more crops to prolong the season in Northwest Vietnam. Intercropping of a native crop that can survive during the dry season may be considered, e.g., we observed some native edible and medicinal species in the field, such as *Streptocaulon juventas* (Lour.) Merr., and *Gymnopetalum cohinchinensis* (Lour.) Kurz. Shade-tolerant crops such as adzuki beans (*Phaseolus calcaratus*) [64] could be introduced as understory crops close to the tree rows. In the fruit-coffee-AF system, light was not used by any crop in the space between coffee rows. An increase in light utilization can be achieved by reducing the distance between coffee rows or intercropping another crop. Although this to some degree hinders coffee management, it may help control weeds and reduce erosion as observed by the local farmers.

## 5 Conclusions

Both agroforestry systems studied utilized more light for biomass production than sole-crop systems and provided stable vegetative cover during the whole year, supporting light interception. Crops on the downslope side of fruit trees were more shaded than upslope crops, particularly close to tree rows. Crops’ yield on the downslope also tended to be lower than in the upslope but showed a significant difference only directly below the tree and grass zones (zone 7). However, maize yield and biomass were only weakly correlated with light distribution. The impact of the trees on light distribution in the fruit tree-crop agroforestry systems varied with tree species, distance and orientation from tree rows, which should be considered when selecting system components and management strategies.

While fruit tree-crop agroforestry utilized incident light more efficiently than sole-crops, thanks to a more stable vegetative cover across the year, there were still available light resources that could be exploited in a system redesign or management plan. The lack of information about favorable light conditions for different annual and/or perennial crops calls for future studies to enable improved agroforestry design and adjustment on sloping land. The slope direction was similar at both sites in this study, so studies are needed on other slope directions. Better knowledge on canopy structures of trees depending on species and management (e.g. pruning strategies) would also benefit species selection and system design.

## Ethics declarations

Review and/or approval by an ethics committee was not needed for this study because its data were collected in field experiments and involved no human participants.

## Funding information

This study was part of the project “Can agroforestry contribute to sustainable development in sloping upland areas of the Mekong region: Project ID 2019-00376” funded by the 10.13039/501100004359Swedish Research Council for Sustainable Development (FORMAS) and the project “Market-based Agroforestry: Potential for Improved Livelihoods, Sustainable Development and Resilience in Smallholder Farming on Sloping Land in the Montane Areas of Mainland Southeast Asia: Project ID 2019-03740” funded by the 10.13039/501100004359Swedish Research Council (VR). Both projects were led by the Swedish University of Agricultural Sciences (SLU) in cooperation with World Agroforestry (CIFOR-ICRAF) and national Soils and Fertilizers Research Institute (SFRI) and provincial Department of Agriculture and Rural Development (DARD) partners in Vietnam.

## Data availability statement

Data will be made available on request.

## CRediT authorship contribution statement

**Huu Thuong Pham:** Writing – review & editing, Writing – original draft, Visualization, Resources, Project administration, Methodology, Investigation, Formal analysis, Data curation, Conceptualization. **Nguyen La:** Writing – review & editing, Supervision, Methodology. **Ingrid Öborn:** Writing – review & editing, Supervision, Methodology, Funding acquisition. **Göran Bergkvist:** Writing – review & editing, Supervision, Methodology. **Rachmat Mulia:** Writing – review & editing, Supervision. **Sigrun Dahlin:** Writing – review & editing, Supervision, Resources, Project administration, Methodology, Funding acquisition.

## Declaration of competing interest

The authors declare that they have no known competing financial interests or personal relationships that could have appeared to influence the work reported in this paper.
