# Supplementary material for: Light distribution at the fruit tree-crop interface and consequences for yield in sloping upland agroforestry
Source: Heliyon. 2024 Sep 29;10(19):e38655. doi: 10.1016/j.heliyon.2024.e38655 (PMC11489345; doi:10.1016/j.heliyon.2024.e38655)
Supplement: Multimedia component 1 [file mmc1.docx]

**Supplementary Materials**

**Table S1:** Management activities in the fruit tree-maize (fruit-maize-AF) agroforestry system and in sole-crop maize (SM) in 2022. Details of fertilizer application are given in Table S3

| **Date** | **Treatment** | **Activity** | **Note** |
| --- | --- | --- | --- |
| 13-Mar-2022 | AF | Fertilizing fruit trees in agroforestry (AF) |  |
| 25-Mar-2022 | AF | Cutting guinea grass in AF. Thinning mango and longan flowers |  |
| 30-Mar-2022 | AF + SM | Hand-hoeing weed in both AF and SM |  |
| 2-May-2022 | AF | Pruning old guinea grass parts to 5 cm height in AF | For better regeneration |
| 10-May-2022 | AF + SM | Hand-hoeing weed second time before sowing maize, in both AF and SM |  |
| 14-May-2022 | AF + SM | Sowing maize in both AF and SM |  |
| 29-May-2022 | AF + SM | Spraying emmabectin benzoate to manage fall army worm on maize, in both AF and SM |  |
| 7-Jun-2022 | AF | Cutting guinea grass in AF |  |
| 8-Jun-2022 | AF | Harvesting mango |  |
| 9-Jun-2022 | AF + SM | Spraying emmamectin benzoate to manage fall army worm on maize, in both AF and SM |  |
| 15-Jun-2022 | AF + SM | Hand-hoeing weed first time for maize First top-dressing fertilizers for maize | Slightly late, because of the need for some sunny days for hand-hoeing |
| 16-Jun-2022 | AF | Fertilizing for fruit tree in AF |  |
| 11-Jul-2022 | AF + SM | Hand-hoeing weed second time for maize. Second top-dressing fertilizer for maize |  |
| 29-Aug-2022 | AF | Harvesting longan |  |
| 18-Sep-2022 | AF + SM | Harvesting maize |  |
| 27-Sep-2022 | AF | Fertilizing for fruit tree in AF |  |
| 29-Sep-2022 | AF | Spraying fipronil to protect spring shoots of both longan and mango |  |
| 15-Oct-2022 | AF + SM | Weeding and cutting maize residues by weed trimmer |  |
| 3-Nov-2022 | AF | Cutting guinea grass |  |
| 4-Dec-2022 | AF | Pruning longan and mango tree |  |

**Table S2:** Management activities in the fruit tree-coffee agroforestry (fruit-coffee-AF) system and in sole-coffee (SC) in 2022. Details of fertilizer application are given in Table S4

| **Date** | **Treatment** | **Activity** | **Note** |
| --- | --- | --- | --- |
| 9-Dec-2021 | AF | Pruning sontra |  |
| 20-Mar-2022 | AF + SC | Weeding 1^st^ time (by strimmer) |  |
| 22-Mar-2022 | AF + SC  AF | Applying fertilizer for coffee, 1^st^ time  Applying fertilizer for fruit trees, 1^st^ time |  |
| 25-Mar-2023 | AF + SC | Spraying acetamiprid and chlopyrifos ethyl to control coffee scale |  |
| 25-Apr-2022 | AF | Cutting guinea grass |  |
| 2-Jun-2022 | AF + SC | Weeding 2^nd^ time (by herbicide) |  |
| 4-Jul-2022 | AF | Cutting guinea grass |  |
| 19-Jul-2022 | AF + SC  AF | Applying fertilizer for coffee, 2^nd^ time  Applying fertilizer for fruit trees |  |
| 10-Sep-2022 | AF | Cutting guinea grass |  |
| Sept-2022 | AF + SC | Harvesting ripe coffee cherries, 1^st^ time |  |
| Sept-2022 | AF | Harvesting sontra fruit |  |
| 1-Oct-2022 | AF + SC | Weeding 3^rd^ time (by strimmer) |  |
| 2-Oct-2022 | AF + SC | Apply fertilizer for coffee, 3^rd^ time  Apply fertilizer for fruit tree |  |
| 14-Oct-2022 | AF + SC | Harvesting coffee cherries, 2^nd^ time |  |
| 17-Nov-2022 | AF + SC | Harvesting coffee cherries, 3^rd^ time |  |
| 15-Dec-2022 | AF + SC | Harvesting coffee cherries, final time. | All remaining cherries (ripe and unripe) |

**Table S3**: Fertilizer types and quantities used in the fruit tree-maize agroforestry (fruit-maize-AF) system in 2022. NPK 6.9.3.8S (6% N, 3.7% P, 2.5% K, 8%S), urea (46%N), KCl (50.6% K), NPK 13.5.10.14S (13% N, 2% P, 8.3% K, 14% S).

| **Date** | **13 Mar 2022** | **14 May 2022** | **15 Jun 2022** | | **12 Jul 2022** | | **27 Sep 2022** | **Total** |
| --- | --- | --- | --- | --- | --- | --- | --- | --- |
| ***Maize (kg/ha)*** | | | | | | | |  |
| *Fertilizer type*  *Quantity* |  | *NPK 6.9.3.8S*  *500* | *Urea*  *175* | *KCl*  *50* | *Urea*  *175* | *KCl*  *50* |  |  |
| N  P  K  S |  | 30  18.4  12.4  40 | 81 | 25.3 | 81 | 25.3 |  | **192**  **18.4**  **63**  **40** |
| ***Fruit tree (kg/tree)*** | | | | | | | |  |
| *Fertilizer type*  *Quantity* | *NPK 13.5.10.14S*  *1.1* |  | *NPK 13.5.10.14S*  *1.1* | |  | | *NPK 5.10.3.8S*  *1.1* |  |
| N  P  K  S | 0.14  0.023  0.091  0.15 |  | 0.14  0.023  0.091  0.15 | |  | | 0.06  0.045  0.025  0.09 | **0.32**  **0.091**  **0.207**  **0.035** |

**Table S4**: Fertilizer types and quantities used in the fruit tree-coffee agroforestry (fruit-coffee-AF) system in 2022. NPK 5.10.3 (6.6% N, 4.1% P, 2.5% K), urea (46%N), KCl (50.6% K), supe phosphate (6.5% P).

| **Date** | **21 Mar 2022** | | | **15 Jun 2022** | | | **12 Oct 2022** | | | **Total** |
| --- | --- | --- | --- | --- | --- | --- | --- | --- | --- | --- |
| ***Coffee (kg/ha)*** |  |  |  |  | |  |  |  |  |  |
| *Fertilizer type*  *Quantity* | *Urea*  *151* | *KCL*  *123* | *Super phosphate*  *313* | *Urea*  *173* | *KCL*  *164* | *Super phosphate*  *313* | *Urea*  *108* | *KCL*  *123* | *Super phosphate*  *313* |  |
| N total  P  K | 70 | 62.3 | 20.5 | 80 | 83 | 20.5 | 50 | 62.3 | 20.5 | **200**  **61.5**  **204.6** |
| ***Sontra (kg/tree)*** |  |  |  |  |  |  |  |  |  |  |
| *Fertilizer type*  *Quantity* | *NPK 5.10.3*  *1.1* | | | *NPK 5.10.3*  *1.1* | | | *NPK 5.10.3*  *1.1* | | |  |
| N  P  K | 0.06  0.045  0.025 | | | 0.06  0.045  0.025 | | | 0.06  0.045  0.025 | | | **0.18**  **0.135**  **0.075** |

**Table S5**: Tree performance in the fruit tree-maize agroforestry (fruit-maize-AF) and fruit tree-coffee agroforestry (fruit-coffee-AF) systems. Tree stem diameter at 10 cm height from ground (D10), tree height, and tree canopy diameter (mean ± standard error). Different letters (a, b) indicate significant differences between longan and mango growth (p=0.05)

| **Parameter** | **Time** | **fruit-maize-AF** | | | **fruit-coffee-AF** | |
| --- | --- | --- | --- | --- | --- | --- |
|  |  | *Longan* | *Mango* | *Sontra* | |  |
| *D10 (cm)* | Mar 2022 | 6.67^a^ ± 0.25 | 11.20^b^ ± 0.42 | 17.71 ± 0.99 | |  |
|  | Jun 2022 | 7.31^a^ ± 0.27 | 12.12^b^ ± 0.48 | 17.00 ± 1.16 | |  |
|  | Sep 2022 | 7.50^a^ ± 0.29 | 12.89^b^ ± 0.54 | 19.06 ± 0.87 | |  |
|  | Dec 2022 | 7.72^a^ ± 0.34 | 12.8^b^ ± 0.50 | 23.29 ± 0.87 | |  |
| *Height (m)* | Mar 2022 | 2.04^a^ ± 0.09 | 3.07 ^b^ ± 0.08 | 5.83 ± 0.16 | |  |
|  | Jun 2022 | 2.08^a^ ± 0.07 | 2.87^b^ ± 0.12 | 5.97 ± 0.15 | |  |
|  | Sep 2022 | 2.06^a^ ± 0.08 | 3.14^b^ ± 0.10 | 6.57 ± 0.15 | |  |
|  | Dec 2022 | 2.16^a^ ± 0.08 | 3.28^b^ ± 0.11 | 7.17 ± 0.17 | |  |
| *Canopy (m)* | Mar 2022 | 2.07^a^ ± 0.10 | 2.80^b^ ± 0.10 | 4.66 ± 0.11 | |  |
|  | Jun 2022 | 2.14^a^ ± 0.10 | 2.53^b^ ± 0.13 | 5.33 ± 0.16 | |  |
|  | Sep 2022 | 2.10^a^ ± 0.13 | 2.82^b^ ± 0.13 | 5.62 ± 0.14 | |  |
|  | Dec 2022 | 2.09^a^ ± 0.09 | 2.78^b^ ± 0.10 | 5.92 ± 0.17 | |  |

**Table S6:** Fraction of light in the fruit tree-maize agroforestry (fruit-maize-AF) and fruit tree-coffee agroforestry (fruit-coffee-AF) systems (mean ± standard error). Different letters (a, b, c) indicate significant differences between sites on four occasions (p=0.05)

| **Experiment** | **Incident light measured on:** | | | |
| --- | --- | --- | --- | --- |
|  | *Mar 2022* | *Jun 2022* | *Sep 2022* | *Dec 2022* |
| *Fruit-maize-AF* | 0.995^ab^ ± 0.002 | 0.989^b^ ± 0.005 | 0.988^a^ ± 0.001 | 0.990^a^ ± 0.001 |
| *Fruit-coffee-AF* | 0.883^c^ ± 0.022 | 0.920^c^ ± 0.015 | 0.838^c^ ± 0.024 | 0.858^c^ ± 0.021 |
| **Effect** | **p-value** | | | |
| Experiment | < 0.001 | | | |
| Time | 0.017 | | | |
| Experiment × Time | 0.021 | | | |


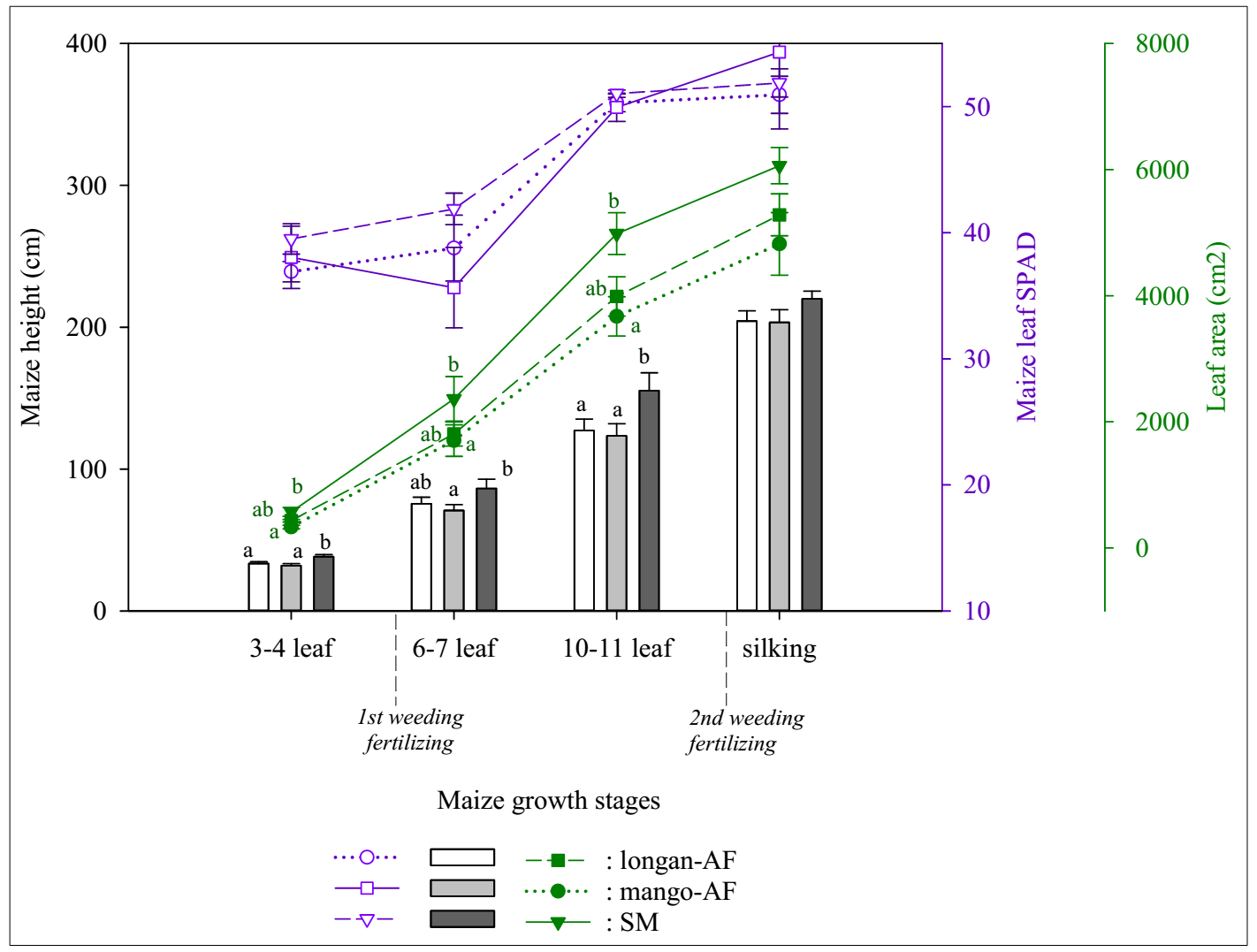


**Figure S1.** Mean maize height, leaf SPAD value, and plant leaf area (LA) in the longan-maize-grass (longan-AF) and mango-maize-grass (mango-AF) agroforestry sub-treatments and in sole-crop maize (SM). Different letters (a, b) indicate significant differences (p=0.05).


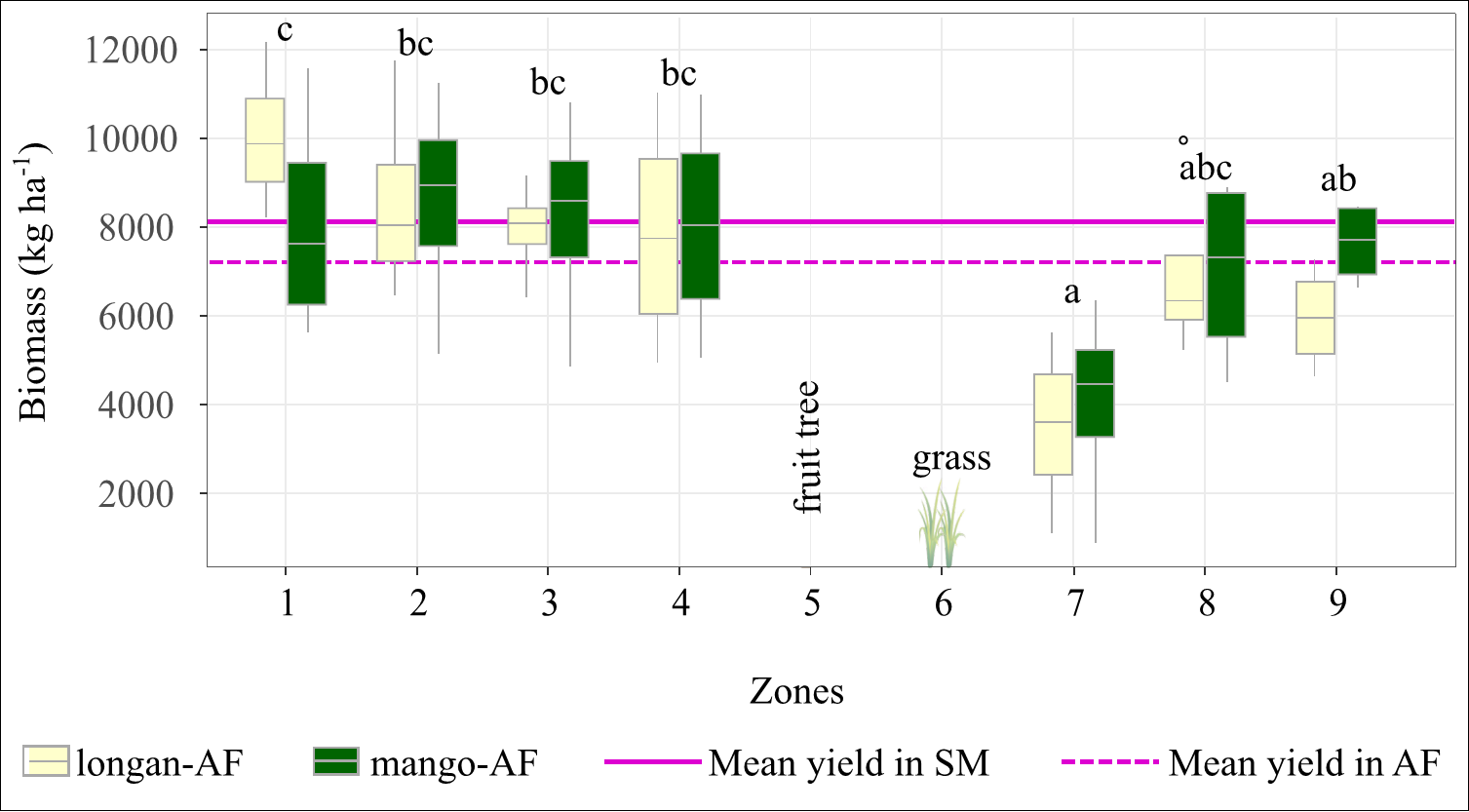


**Figure S2.** Maize aboveground biomass in crop zones in longan-maize (longan-AF) and mango-maize (mango-AF) agroforestry sub-treatments. Main effect of zone was significant (p<0.001). Different letter*s* (a<b) indicate significant differences between maize zones (p=0.05). Error bars show 95% confidence interval.


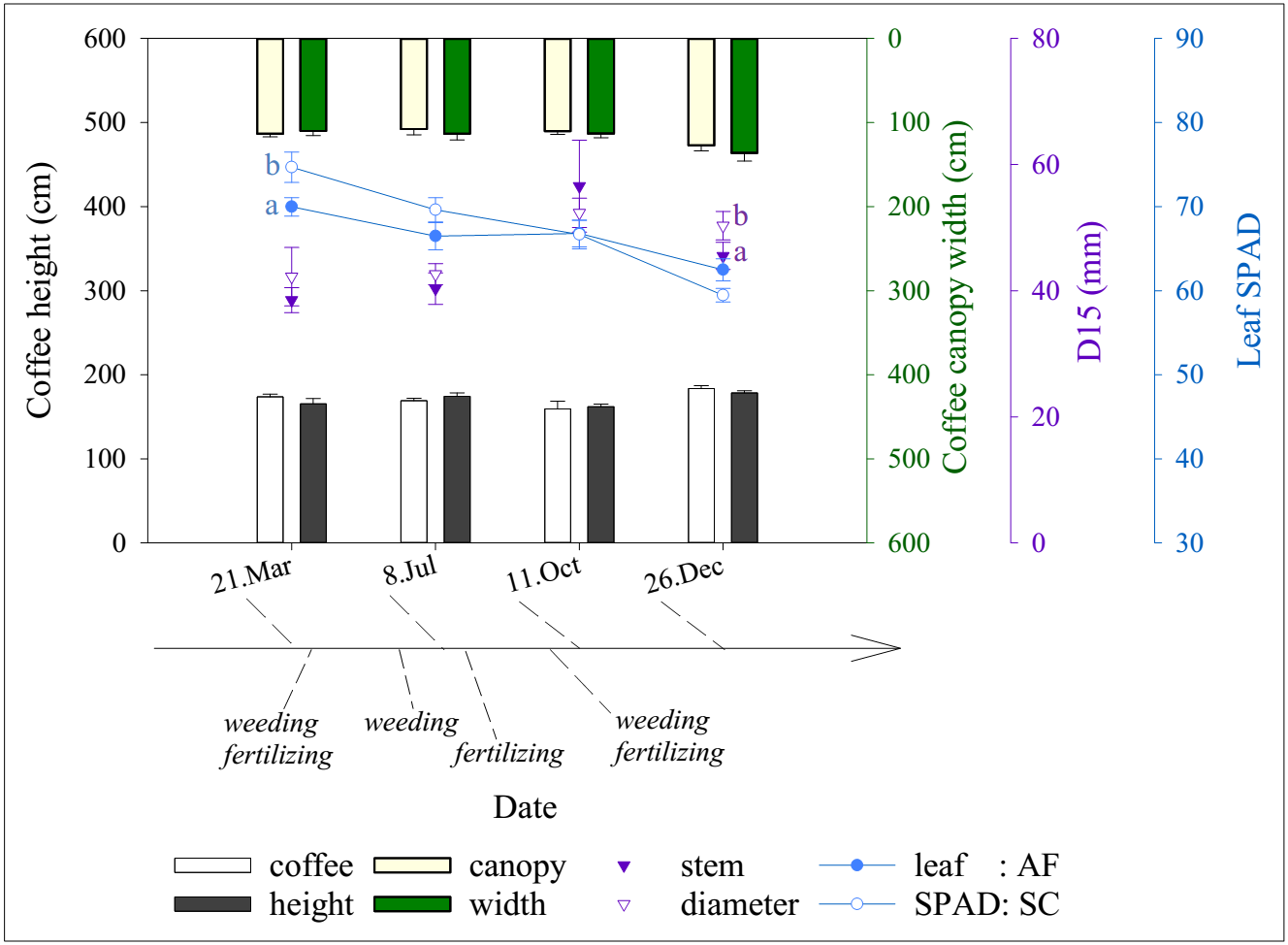


**Figure S3***.* Coffee growth performance in agroforestry (AF) and sole-coffee (SC) treatments in fruit tree-coffee agroforestry (fruit-coffee-AF) experiment in 2022. Different letters (a, b) indicate significant differences (p=0.05) between treatments on each measurement occasion.


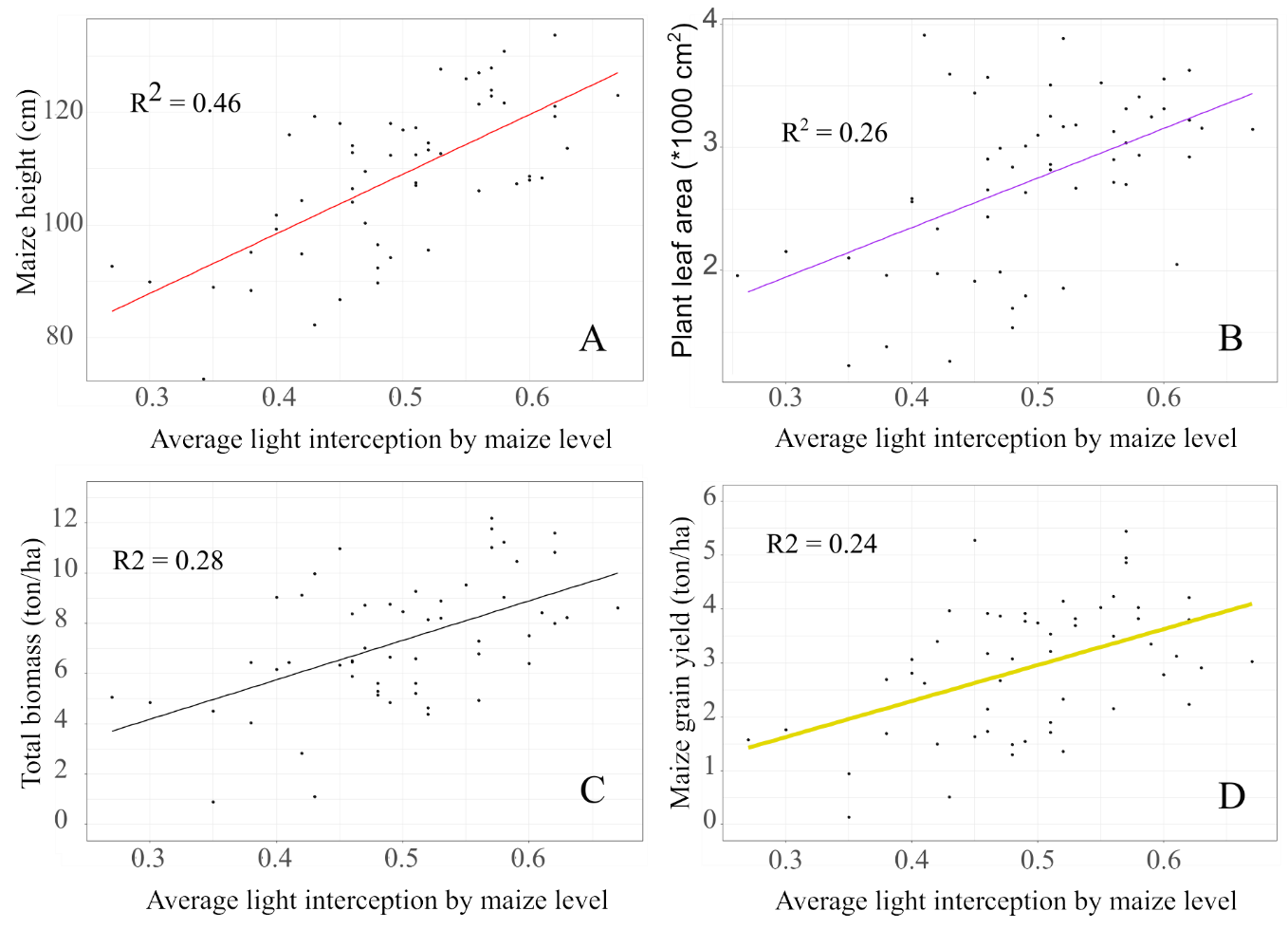


**Figure S4**. Correlation between average light interception by maize level and (a) maize height, (b) plant leaf area, (c) total aboveground biomass and (d) grain yield in fruit tree-maize agroforestry (fruit-maize-AF) in 2022.
